# Increasing the perceived relevance of cervical screening in older women who do not plan to attend screening

**DOI:** 10.1136/sextrans-2019-054120

**Published:** 2019-08-08

**Authors:** Laura A V Marlow, Mairead Ryan, Jo Waller

**Affiliations:** Department of Behavioural Science and Health, University College London, London, UK

**Keywords:** risk perceptions, non-attenders, older, cervical screening, pap smear, salience, timeline

## Abstract

**Objectives:**

Uptake of cervical screening among women aged 50–64 years is declining. Not feeling at risk because of current sexual behaviour is a reason some older women give for not being screened. We hypothesised that explaining the long interval between acquiring human papillomavirus (HPV) and developing cervical cancer would increase the relevance of screening in older women.

**Methods:**

Women aged 50–64 years (n=597) who did not intend to go for screening were recruited through an online panel and randomised to one of three information conditions: *cause only* (basic information about HPV and cervical cancer), *cause with basic timeline* (also read a sentence describing the long interval between acquiring HPV and developing cervical cancer) and *cause with explicit timeline* (read the same as the timeline group alongside an explanation of what this means for older women). Perceived risk of cervical cancer, screening intention strength and understanding of HPV were assessed preinformation and postinformation exposure.

**Results:**

Information condition was significantly associated with risk perceptions and intention strength postintervention (F(2,593)=6.26, p=0.002 and F(2,593)=4.98, p=0.007 respectively). Women in the *cause with explicit timeline* condition were more likely to increase their risk perceptions and intention strength compared with *cause only* (24% vs 9% and 25% vs 13% for risk perceptions and intention, respectively). In the *cause with explicit timeline* group, women with 4–10 lifetime partners had higher odds of increasing their perceived risk and intention strength postintervention compared with those with 0–1 partners (OR=2.27, 95% CI 1.01 to 5.12 and OR=3.20, 95% CI 1.34 to 7.67, respectively).

**Conclusions:**

Providing a clear explanation that decouples women’s perceived cervical cancer risk from their current sexual behaviour has the potential to increase perceived risk of cervical cancer and intentions to be screened among older women. Providing women with a clear cognitive representation of the aetiology of cervical cancer may be one approach to increasing screening uptake.

## Introduction

Adequate cervical screening between 50 and 64 years is associated with lower risk of cervical cancer,^1^ yet a growing number of women in this age range are not attending screening.^2^ Recent work suggests that unlike younger women who are more likely to intend to go for screening but not get around to it, older women are more likely to have made an active decision not to be screened.^3^ Further exploration of health beliefs in the same sample suggests that women who decide not to be screened have lower cervical cancer risk perceptions.^4 5^ Older women are also less likely to believe that cervical screening reduces their risk of cancer^6^ and some studies suggest that perceived relevance of screening is lower in older women because of perceptions about their current sexual activity.^2 7 8^

Having many sexual partners is the most commonly mentioned risk factor when using unprompted questions about what causes cervical cancer,^9^ and older women are more likely to cite sexual activity and having multiple partners as causes of cervical cancer than younger women.^6^ The introduction of human papillomavirus (HPV) primary screening and associated shift in the focus of screening to look for an STI may increase awareness of this association.^7^ In a study where women were asked to read information about HPV, its sexually transmitted nature and its link with cervical cancer, perceived risk of cervical cancer increased among the youngest women (<25 years) but decreased among the oldest women (>64 years).^10^ Perceived risk did not change for women who were in the screening age range (25–64 years).

Evidence suggests that women over 50 years are less likely to have new sexual partners and more likely to be in long-term monogamous relationships,^11^ but progression from persistent HPV to cervical cancer takes an average of 12–15 years, so previous, rather than current, sexual behaviour is indicative of risk.^12^ A newly detected HPV infection in an older woman could result from an infection acquired many years ago, reflecting persistence or reactivation, rather than a new infection.^13^ Most information about cervical cancer now incorporates details of the causal role of HPV; however, a systematic review of studies exploring women’s frequently asked questions about HPV suggested that many have a desire to understand the ‘timeline’ of progression from HPV to cervical cancer.^14^ In addition, Leventhal’s common sense model of illness^15^ theorises that beliefs about causes and timeline, as well as consequences, control and symptoms are important in helping people develop a coherent cognitive representation of their illness. This provides a useful framework for considering aspects of HPV that should be communicated to women to help improve their understanding of HPV and its link with cervical cancer.^16^ For older women in particular, good integration of timeline and causal information would facilitate a more coherent understanding of their risk, by making it clear that previous sexual history is as important as current sexual behaviour. The present study tested the hypothesis that presenting information about the long timeline of progression from HPV to cervical cancer could increase perceived risk of cervical cancer and cervical screening intention strength among women aged 50–64 years.

## Methods

### Participants

Participants were identified through an online panel maintained by Dynata Global UK ltd. Emails were sent to participants who were registered as being female and aged 50–64 years. The email asked participants to click a link, which directed them to an online survey (hosted by Survey Monkey). After reading the study information page and indicating consent to participate, women were asked to confirm their age, country of residence and their future screening intentions. Women were eligible for the study if they were 50–64 years old, living in Britain and responded that they would ‘probably not’ or ‘definitely not’ attend cervical screening the next time they were invited (ie, they were non-intenders). We estimated that 200 women in each condition would result in approximately 80% power to detect a difference of 0.22 in perceived risk of cervical cancer (SD=0.8) with alpha=0.05. The expected effect size was based on a previous study exploring perceived risk of cervical cancer preinformation and postinformation about HPV.^17^ We therefore commissioned recruitment of 600 non-intenders. Recruitment emails were sent in waves until the target sample size had been reached.

### Procedure

Eligible women completed baseline measures assessing perceived risk of cervical cancer, cervical screening intention strength and attitudes to screening. Participants were then randomised to read one of three pieces of text about HPV and cervical cancer. After reading the information, women were asked to complete three questions based on its content, as a comprehension check. If any of these were incorrect, they were returned to the information and asked to read it again. The comprehension check was repeated up to two times before letting a participant progress. Participants then completed the items on perceived risk, intention strength and attitudes to screening for a second time.

### Intervention

The intervention was designed to improve knowledge about the timeline of HPV and cervical cancer, a gap in knowledge that has been identified in previous studies.^14^ This intervention focuses on the ‘education’ function of the Behaviour Change Wheel,^18^ and we hypothesised this would increase women’s motivation to attend. Participants were randomised to one of three information conditions (see table 1): *cause only* (basic information about HPV and cervical cancer), *cause with basic timeline* (also read a sentence describing the long interval between acquiring HPV and developing cervical cancer) and *cause with explicit timeline* (read the same as the timeline group alongside an explanation of what this means for older women).

**Table 1:** Information provided to women

**Cause only information (control**)^*^	*What affects my chances of getting cervical cancer?* Almost all cases of cervical cancer are caused by HPV infections. HPV can be passed on through any type of sexual activity with a man or women. Women and men who have had more sexual partners are more likely to get HPV infections. However, HPV is so common that most people will have the virus at some point in their life. HPV is found on the skin around the whole genital area and can spread through any type of sexual activity. This means that condoms do not protect you from getting an HPV infection.
**Cause with basic timeline information**	Same information as the control information, followed by: HPV can take a long time to develop into cancer (10–30 years).
**Cause with explicit timeline information**	Same information as the control information, followed by: Women aged 50–64 years should be aware that HPV can take a long time to develop into cancer (10–30 years). This means that even if you have not been sexually active for a long time or have only had one partner for a long time, you could still be at risk of cervical cancer.

*This information is from the NHS cervical screening programme ‘Helping you decide’ leaflet.

HPV, human papillomavirus; NHS, National Health Service.

### Measures

Outcomes: perceived risk of cervical cancer was assessed using a single item: ‘How likely do you think you are to develop cervical cancer in the next 10 years?’. Cervical screening intention strength was assessed using the question: ‘How likely is it that you will attend screening when next invited?’. Both outcome measures used a 7-point Likert scale (from very unlikely to very likely). A single item assessed self-rated understanding of HPV ‘I understand how human papillomavirus (HPV) can cause cervical cancer’ using a 7-point Likert scale (from strongly disagree to strongly agree).

Sociodemographic characteristics: age was collected using an open box at the beginning of the survey. At the end of the survey, participants were asked additional sociodemographic questions including ethnicity (17 categories – based on the 2011 census question),^19^ highest educational qualification (no formal qualifications, O-levels or General Certificate of Secondary Education (GCSE) or equivalent, A-levels, Ordinary National Certificate (ONC)/Business and Technology Education Council (BTEC)/National Vocational Qualification (NVQ), degree or higher, still studying and don’t know) and marital status (single, married/cohabiting, separated, divorced and widowed). Participants were also asked if they had heard of HPV (yes, no and don’t know).

Sexual history: women were asked three questions about their sexual history based on the National Survey of Sexual Attitudes and Lifestyles questionnaire (Natsal-3).^20^ These included whether they were currently in a sexual relationship (yes or no); number of previous partners (0, 1, 2–3, 4–5, 6–10, 11–20 and 20+) and length of time since last new partner (within the last 1 year, 5 years, 10 years, 20 years and over 20 years). All sexual history items included a ‘prefer not to say’ option.

### Analysis

Primary analyses: we used analyses of covariance (ANCOVA) to establish if there were main effects for information condition (three levels: *cause only*, *cause with basic timeline* and *cause with explicit timeline*) on perceived risk of cervical cancer and cervical screening intention strength (adjusting for baseline scores). To explore the differences in more detail, change scores were calculated by subtracting preintervention scores from postintervention scores and reclassifying responses into binary outcomes for change in risk perception and change in intention (1=increased; 0=decreased/no change). This allowed us to look at the proportion of women in each condition who increased their perceived risk and intention strength. Percentage change, χ^2^ (with p value) and OR, with CI are reported. Sensitivity analyses were used to see if excluding participants who failed the comprehension check twice (n=92) influenced the findings.

Secondary analyses: we initially planned (a priori) to explore associations between marital status/sexual history and change in intention in the intervention groups to see if these variables moderated any impact of the intervention. Since the *cause with basic timeline* intervention did not have any overall impact, we decided to look only at women in the *cause with explicit timeline* group (n=216). We ran logistic regression models to explore bivariate associations between marital status and sexual history (current sexual relationship, number of lifetime partners and time since last new partner) and having increased perceived risk or screening intention strength (reference group=those who decreased or did not change their risk/intention). ORs with CI are reported. We also explored whether the interventions improved self-rated understanding of HPV using ANCOVA (adjusting for baseline scores).

## Results

### Sample

In total, 4215 eligible women aged 50–64 years were directed to the survey and consented to participate. Fifteen per cent of these women said they would probably not (n=450) or definitely not (n=173) attend cervical screening when next invited and were included in the study (see online supplementary figure 1). Sample characteristics are reported in table 2. Most women were currently overdue for screening (n=367) and 18% reported that they had never been screened (n=105). Half of the women were married and over a third had degree level education (40%). Most women reported that they were not currently in a sexual relationship (62%) and very few reported having a new partner within the last 5 years (5%), but 42% reported four or more lifetime sexual partners. Women were randomised to the three conditions as follows: *cause only* (n=183), *cause with basic timeline* (n=198) or *cause with explicit timeline* (n=216). There were no sociodemographic or sexual history differences between women in the three conditions and no differences in the number of times women read the information they were provided by group.

10.1136/sextrans-2019-054120.supp1 Supplementary data

**Table 2:** Sample characteristics (n=597)

	n	%
Age (years)		
50–54	171	28.6
55–59	180	30.2
60–64	246	41.2
Education		
No formal qualifications	29	4.9
Any below degree	312	52.3
Degree	237	39.7
Other/still studying	19	3.2
Ethnicity		
White British	551	92.3
Non-white British	40	6.7
Marital status		
Married or cohabiting	314	52.6
Separated, divorced, widowed	121	20.3
Single	155	26.0
Heard of HPV		
Yes	433	72.5
No/don’t know	164	27.5
Currently in a sexual relationship		
Yes	197	33.0
No	370	62.0
Prefer not to say	30	5.0
Number of partners over the lifetime		
None	40	6.7
1	135	22.6
2–3	123	20.6
4–10	158	26.5
>10	90	15.1
Prefer not to say	50	8.4
Last new partner		
Within the last 10 years	105	17.6
Within the last 20 years	99	16.6
20 years or more	332	55.6
Prefer not to say	58	9.7
Screening status		
Up to date	125	20.9
Overdue	367	61.5
Never had a test	105	17.6

Note: percentages that do not add up to 100% are due to missing data.

HPV, human papillomavirus.

### Perceived risk and intention strength

Information condition was significantly associated with risk perceptions (F(2,593)=6.26, p=0.002; *cause only* $\bar{x}$=1.72, *cause with basic timeline* $\bar{x}$ =1.82 and *cause with explicit timeline* $\bar{x}$=1.98) and intention strength (F(2,593)=4.98, p=0.007; *cause only* $\bar{x}$=2.15, *cause with basic timeline* $\bar{x}$=2.16 and *cause with explicit timeline* $\bar{x}$=2.30). In sensitivity analyses excluding women who failed the comprehension check, the means and SD were similar and differences remained significant.

**Figure 1:**
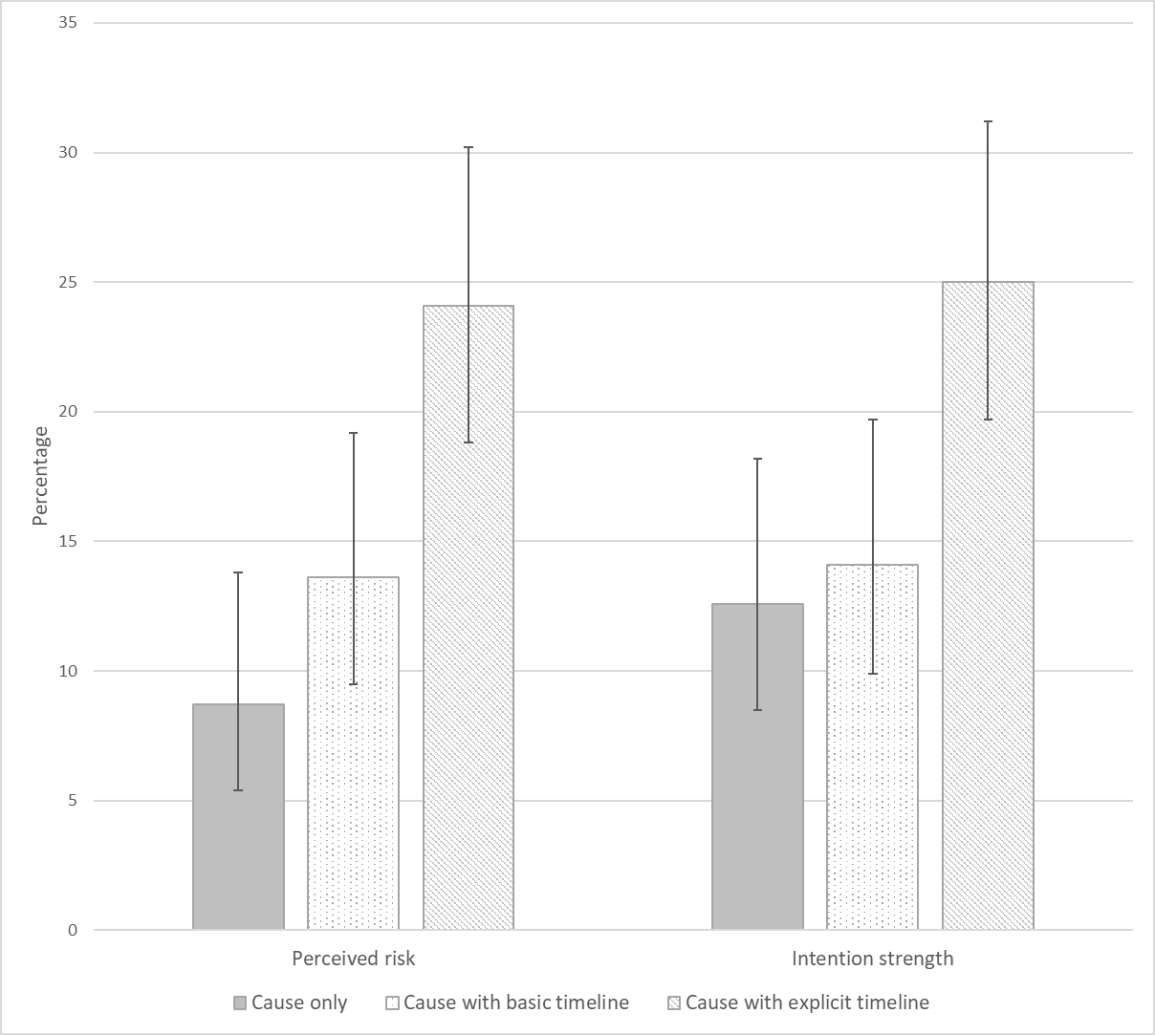
Percentage of women who increased their perceived risk and intention strength after reading the information provided (with 95% CIs).

Figure 1 shows the percentages of women whose perceived risk increased after reading the information provided. Women in the *cause with explicit timeline* condition were more likely to have an increase compared with those in the *cause only* condition: 24% v 9%, (χ^2^(1)=16.47, p<0.001; OR=3.31, 95% CI 1.82 to 6.03) and were also more likely to increase their intention strength: 25% versus 13% (χ^2^(1)=9.83, p<0.001; OR=2.32, 95% CI 1.36 to 3.96). The percentages of women with increases in perceived risk and intention strength in the *cause with basic timeline* condition were no different from the *cause only* condition (14% vs 9%: χ^2^(1)=2.27, p=0.132; OR=1.65, 95% CI 0.86 to 3.17 and 14% vs 13%: χ^2^(1)=0.20, p=0.652; OR=1.15, 95% CI 0.63 to 2.07, respectively).

### Marital status and sexual history

Among women in the *cause with explicit timeline* condition (n=216), we explored whether marital status or sexual history were associated with increasing risk perceptions or intention strength after reading the information provided (see table 3). Women who had 4–10 lifetime partners were significantly more likely to increase their perceived risk and intention strength postintervention, compared with those with 0–1 sexual partner. There was no significant association for marital status, current sexual relationship status or length of time since last partner.

**Table 3:** Unadjusted odds of increased risk/intention among women receiving cause with explicit timeline information (n=216)

	Increased risk perceptions		Increased intention strength	
	%	OR (95% CI)	%	OR (95% CI)
Marital status				
Married or cohabiting (n=122)	23.0	1.00	21.3	1.00
Separated, divorced, widowed (n=43)	25.6	1.15 (0.52 to 2.58)	34.9	1.98 (0.92 to 4.24)
Single (n=48)	27.1	1.25 (0.58 to 2.68)	25.0	1.23 (0.56 to 2.70)
Currently in a sexual relationship				
No (n=126)	26.2	1.00	23.8	1.00
Yes (n=78)	23.1	0.85 (0.44 to 1.64)	25.6	1.10 (0.57 to 2.12)
Number of partners over the lifetime				
0–1	18.8	1.00	14.1	1.00
2–3	20.5	1.11 (0.43 to 2.29)	25.0	2.04 (0.76 to 5.43)
4–10	34.4	2.27 (1.01 to 5.12)*	34.4	3.20 (1.34 to 7.67)**
>10	14.3	0.72 (0.21 to 2.47)	21.4	1.67 (0.53 to 5.24)
Last new partner				
Within the last 10 years	16.7	1.00	22.2	1.00
Within the last 20 years	34.3	2.61 (0.85 to 8.00)	34.3	1.83 (0.64 to 5.22)
20 years or more	22.0	1.41 (0.53 to 3.73)	22.0	1.05 (0.40 to 2.41)

*P<0.05, **p<.01.

### Self-Rated understanding of HPV

Preintervention, 54% of women agreed that they understood how HPV can cause cervical cancer; postintervention this was 88%. There were no between-group differences in self-rated understanding of HPV after reading the information provided: F(2,593)=0.79, p>0.05.

## Discussion

Reading brief information about the role of HPV in cervical cancer aetiology can improve women’s confidence in their understanding of the causal pathway and is important for the move to HPV primary screening. For older women, explaining the long interval between acquiring HPV and developing cervical cancer can increase risk perceptions and intentions to be screened. However, providing a clear explanation for what the time lag means is important. Shifting risk perceptions and intentions to be screened is notoriously difficult, so the changes seen here are encouraging, especially because the women we included had all indicated that they probably or definitely would not go for screening in the future. In addition, the greater shifts observed among women whose sexual history placed them at greater risk was encouraging. National Health Service leaflets are currently being updated to take account of the move to HPV primary screening and development of an evidence-base to inform decisions about which information to include is important. Small changes to wording of written health information are low cost and easy to implement so even if effects are small, making changes to information materials based on evidence about which messages are beneficial is likely to be cost-effective.

Providing information about the timeline from HPV acquisition to cervical cancer development, in line with psychological theory,^15 16^ can help women to form a more integrated cognitive representation of this process. This is important to make sure those who have decided not to be screened are making informed decisions. Developing targeted cervical screening information for older women may be the most helpful way to disseminate this message. Targeting materials to segments of the population can help to relieve the burden of sifting through unnecessary information and also address in greater detail factors that are relevant to that specific sub-group.^21^

### Strengths and limitations

Participants were recruited through an online panel which means they do not necessarily represent the general population. This did however mean that we were able to selectively recruit women who did not intend to go for screening in the future, a group who are difficult to recruit through other routes. The proportion of women in our sample reporting more than 10 sexual partners was broadly similar to findings from Natsal (15% in our sample and 12% in Natsal) but women reporting no sexual partners were over-represented (as would be expected, as cervical screening uptake is low in women who have never been sexually active). Item non-response for the sexual history items was 5%–10%, which was slightly higher than in Natsal but lower than similar questions asked in the Health Survey for England 2010.^22^ The format of the study meant that women were asked to read a piece of information online and asked to reread this if they got the comprehension check wrong. While this allowed us to compare the impact of different information in a highly controlled way, it lacks the ecological validity that would come from testing the impact of this information when embedded in an information leaflet arriving alongside a screening invitation, which women may or may not read. The ‘real-world’ impact of such information would therefore need further support, but proof of principle research can be helpful in determining evidenced-based message content worthy of further testing in health materials.^23^

The mean overall change on the seven-point scale that was used to assess intention was from 2.1 to 2.3, suggesting that across the sample the majority of women would still not intend to go for screening (responses of <3 represent a response of ’unlikely’). This is perhaps not surprising given that these women were non-intenders to begin with. A quarter of women reading the timeline information increased their intentions to be screened after the intervention, and while this is positive, it needs to be interpreted with caution. For some of these women, the intention shift was from selecting ‘very unlikely’ to ‘unlikely’. While this is technically an increase in intention, it may not be considered a meaningful shift since intentions are still negative. In addition, given consistent evidence of an intention–behaviour gap in screening behaviour, further attenuation would be expected in the translation to behaviour. It is therefore likely that the intervention we have used would result in far fewer than a quarter of non-intenders getting screened.

The intervention developed here falls within the ‘education’ function of the Behaviour Change Wheel: increasing knowledge and understanding of HPV and cervical cancer.^18^ While information about the HPV–cervical cancer timeline may help increase motivation to be screened, interventions will need to be multifaceted in order to overcome other types of barriers that women report in relation to cervical screening (eg, emotional or physical discomfort or being too busy to attend). Making changes to the way in which cervical screening is communicated and offered will hopefully address barriers across all aspects of the behaviour system (capability, opportunity and motivation) and accumulate to have a significant impact on screening participation. Such changes may include incorporating effective cues to action alongside well-developed information. This could include prompts to plan participation, for example, using implementation intentions^24^ or system level changes (eg, offering preset appointments or using textmessage reminders). Though this study tested the impact of providing a written message about the relevance of screening for older women, the same message could become part of a face-to-face provider-led intervention for older women who do not wish to be screened. This may be particularly helpful in countries like the USA where postal invitations are less routine.

### Conclusion

For older women, explaining the long interval between acquiring HPV and developing cervical cancer alongside an explicit explanation for what the time lag means for women their age has the potential to increase cervical screening intention strength in those with low screening intentions. Ideally, information for older women would provide this message in a targeted way. Considering further ways to improve the accuracy of women’s cognitive representations of HPV and cervical cancer may help facilitate informed screening uptake.

**Key messages**

- Not feeling at risk of cervical cancer because of current sexual behaviour is one reason older women give for not being screened.
- A newly detected human papillomavirus (HPV) infection in an older woman could result from an infection acquired many years ago.
- Explaining the long interval between acquiring HPV and developing cervical cancer can increase the relevance of screening in older women.
