# Supplementary material for: Increasing the perceived relevance of cervical screening in older women who do not plan to attend screening
Source: Sex Transm Infect. 2019 Aug 8;96(1):20–5. doi: 10.1136/sextrans-2019-054120 (PMC7029243; doi:10.1136/sextrans-2019-054120)
Supplement: Supplementary data [file sextrans-2019-054120supp001.pdf]

Supplementary Figure 1: Recruitment flow diagram

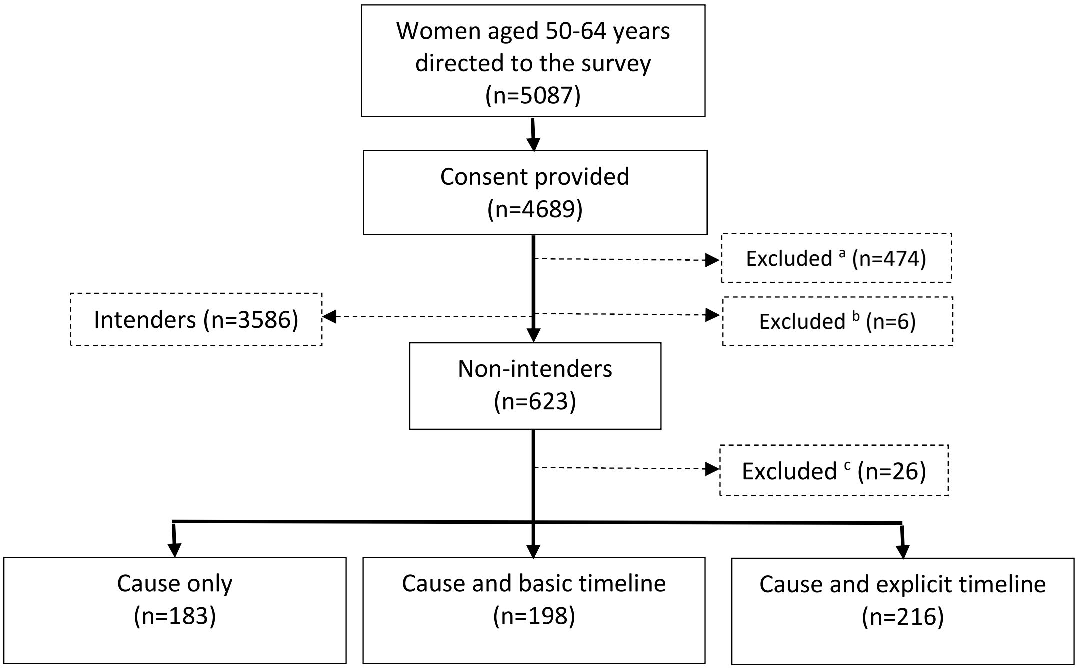

[a] Never heard of screening, never been invited, had a hysterectomy
[b] Intention question not answered
[c] Dropped out
